# Human Amnion Epithelial Cells Induced to Express Functional Cystic Fibrosis Transmembrane Conductance Regulator

**DOI:** 10.1371/journal.pone.0046533

**Published:** 2012-09-28

**Authors:** Sean V. Murphy, Rebecca Lim, Philip Heraud, Marian Cholewa, Mark Le Gros, Martin D. de Jonge, Daryl L. Howard, David Paterson, Courtney McDonald, Anthony Atala, Graham Jenkin, Euan M. Wallace

**Affiliations:** 1 The Ritchie Centre, Monash Institute for Medical Research, Monash University, Melbourne, Australia; 2 Centre for Biospectroscopy, School of Chemistry, Monash University, Melbourne, Australia; 3 Monash Centre for Synchrotron Science, Monash University, Melbourne, Australia; 4 Physical Biosciences Division, Lawrence Berkeley National Laboratory, Berkeley, California, United States of America; 5 Australian Synchrotron, X-ray Fluorescence Microscopy, Melbourne, Australia; 6 Department of Obstetrics and Gynaecology, Monash University, Melbourne, Australia; 7 Wake Forest Institute for Regenerative Medicine, Wake Forest University School of Medicine, Winston-Salem, North Carolina, United States of America; University of Pittsburgh, United States of America

## Abstract

Cystic fibrosis, an autosomal recessive disorder caused by a mutation in a gene encoding the cystic fibrosis transmembrane conductance regulator (CFTR), remains a leading cause of childhood respiratory morbidity and mortality. The respiratory consequences of cystic fibrosis include the generation of thick, tenacious mucus that impairs lung clearance, predisposing the individual to repeated and persistent infections, progressive lung damage and shortened lifespan. Currently there is no cure for cystic fibrosis. With this in mind, we investigated the ability of human amnion epithelial cells (hAECs) to express functional CFTR. We found that hAECs formed 3-dimensional structures and expressed the *CFTR* gene and protein after culture in Small Airway Growth Medium (SAGM). We also observed a polarized CFTR distribution on the membrane of hAECs cultured in SAGM, similar to that observed in polarized airway cells *in vivo.* Further, hAECs induced to express CFTR possessed functional iodide/chloride (I^−/^Cl^−^) ion channels that were inhibited by the CFTR-inhibitor CFTR-172, indicating the presence of functional CFTR ion channels. These data suggest that hAECs may be a promising source for the development of a cellular therapy for cystic fibrosis.

## Introduction

The need for a cure of cystic fibrosis remains as urgent today as ever. Since the discovery of the *CFTR* gene and the mutations that cause cystic fibrosis, attempts at gene correction by *CFTR* gene transfer to the airways, using both viral and non-viral gene-transfer mechanisms, have been described [1], [2], [3]. Unfortunately, this approach to treatment, at least to date, has been unsuccessful. Delivery of viral vectors to patients with cystic fibrosis is inefficient [4], [5] and even when successfully delivered, the resultant normal *CFTR* expression is only transient and does not result in long-term functional improvement [6], [7]. An alternative approach could be a cell-based therapy using cells with functional CFTR that integrate into the respiratory tract.

We have shown that placental-derived, human amnion epithelial cells (hAECs) offer promise for such a therapy. Cells isolated from the human placenta and fetal membranes have considerable advantages over other sources of stem cells such as embryonic or bone marrow-derived cells. They are plentiful, multipotent, immunosuppressive, have a low immunogenic profile, lack tumorigenicity, and present no ethical barriers to their collection [8], [9]. With a view to developing a possible therapy for lung disorders, we have shown that hAECs can be differentiated *in vitro* toward a respiratory-like phenotype [8], [10]. Further, in a mouse model of acute lung injury hAECs will engraft in the lung in significant numbers, constituting approximately 5% of total lung cells, with over 70% of engrafted cells differentiating *in vivo* towards a pulmonary phenotype [10]. Here we report the ability to induce hAECs to express functional CFTR *in vitro*, providing a valuable tool for a potential cellular therapy for cystic fibrosis.

## Materials and Methods

### Isolation and Culture of Human Amnion Epithelial Cells (hAECs)

The collection and use of human tissues were undertaken with informed, written consent and with approval of Southern Health Human Research and Ethics Committee and the Monash University Human Ethics Committee (Approval No. 01067B: Establishment of a tissue bank and associated database for use by the Centre for Women’s Health Research). Human amnion epithelial cells (hAECs) were isolated as previously described [8]. Briefly, placentae were obtained from women with an uncomplicated pregnancy undergoing an elective caesarean section at term (38–40 weeks’ gestation). Placentae were washed with sterile normal saline to remove blood and the amnion membrane manually stripped from the chorion. Epithelial cells were enzymatically removed from the amnion by two 1 hour digestions in Trypzean at 37°C (Sigma-Aldrich, St Louis, MO, USA). Epithelial cells were collected by centrifugation and Trypzean was inactivated by resuspending cells in sterile normal saline containing 1 mg/ml soybean trypsin inhibitor (Sigma-Aldrich, St Louis, MO, USA). hAECs were cultured in either Small Airway Epithelial Growth Medium (SAGM) (Lonza Australia Pty Ltd, Mt Waverley, VIC, Australia) or DMEM/F12 with 10% FBS for up to 28 days without passage.

### Nucleic Acid Purification

Total RNA was isolated using TRIZOL® reagent according to manufacturer’s instructions (Invitrogen, Mulgrave, VIC, Australia). RNA concentration was measured on Nanodrop (Thermo Fisher Scientific, North Ryde, NSW, Australia) and used immediately for polymerase chain reaction (PCR). Complementary DNA (cDNA) was synthesized from 500 ng total RNA in a final reaction volume of 20 µL using Thermoscript Reverse Transcription System (Invitrogen). After denaturing the template RNA, 10 mM dNTP Mix and Oligo (dT)_20_ primers at 65°C for 5 minutes, 15U of ThermoScript™ Reverse Transcriptase was added in the presence of 5X cDNA Synthesis Buffer (250 mM Tris acetate (pH 8.4), 375 mM potassium acetate, 40 mM magnesium acetate, stabilizer), 0.1 M Dithiothreitol (DTT), 40 U RNaseOUT™ and RNase-free water to complete the final volume. The reaction mixture (20 µL) was incubated at 60°C for 1 h and then stopped at 85°C for 5 minutes.

### Quantitative RT-PCR

Quantitative RT-PCR analysis was performed with the TaqMan® Gene Expression Assay (Hs00357011_m1) targeted against CFTR, and (Hs03928990_g1) targeted against 18 S (Invitrogen), using the Applied Biosystems 7900HT fast real time PCR system (Applied Biosystems, Mulgrave, VIC, Australia). Human lung total RNA (Ambion, Invitrogen) was used as a positive control sample for this study. Quadruplicate 20 µL reactions were performed for both CFTR and 18 S. CFTR gene expression was normalized to the 18 S endogenous control to account for variability in the initial concentration and quality of the total RNA and in the conversion efficiency of the reverse transcription reaction. Results were analysed as per manufacturer’s instructions. Briefly, Sequence Detection System (SDS) software was used to automatically determine the baseline and threshold for the amplification curves. Relative quantitation was performed using the comparative CT method, comparing a CFTR gene expression against an internal calibrator sample from day 0. CFTR gene expression was expressed as a fold change from this internal calibrator sample. This was achieved by dividing the normalized test sample by the normalized calibrator sample.

### Western Blot

Whole cell extracts were electrophoresed in polyacrylamide gels and transferred onto nitrocellulose membranes. Membranes were blocked with blocking buffer (5% w/v non-fat dry milk, 1X TBS, 0.1% Tween-20) followed by incubation with human CFTR antibody (#MAB25031) at 1 µg/mL (R&D Systems, Minneapolis, MN, USA) incubated overnight at 4°C. Membranes were then incubated with anti-mouse IgG-HRP secondary antibody (Cell Signaling #7076, Invitrogen) at 1∶10000 for 1 hr at room temperature. Bands were visualised using an Immobilon™ Western Chemiluminescent HRP substrate (Millipore, Billerica, MA, USA). Nitrocellulose membranes were stripped using mild stripping buffer, blocked, and incubated with mouse anti-β-actin primary antibody (#A5441) at 1∶5000 (Sigma-Aldrich, St Louis, MO, USA) overnight at 4°C. Membranes were then incubated with anti-mouse IgG-HRP secondary antibody at 1∶10000 (Cell Signaling #7076, Invitrogen) for 1 hr at room temperature. Bands were visualised as described above.

### Flow Cytometery

Human amnion epithelial cells were fixed in 4% paraformaldehyde in PBS for 15 minutes at 4°C and were permeabilized with 0.1% Triton-X-100 in PBS for 5 minutes at room temperature. DAKO Protein Block Serum-Free (Dako Australia Pty Ltd, Botany, NSW, Australia) was used to block non-specific binding for 10 minutes at room temperature and cells incubated in 8 µg/mL human CFTR antibody (#MAB25031, R&D Systems) diluted in DAKO antibody diluent for 1 hour at room temperature. Alexa Fluor 488 goat anti-mouse IgG (#A-11001, Life Technologies, Mulgrave, VIC, Australia) was used as a secondary antibody at 40 µg/mL for 1 hour at room temperature. Mouse IgG2A Isotype Control (#MAB0031, R&D Systems) was used for isotype control samples. Samples were analysed with the BD Biosciences FACSCalibur flow cytometer (BD Australia, North Ryde, NSW, Australia).

### Immuno-fluorescent Staining and Imaging

To image 3D structures formed by hAECs cultured in SAGM, cells were stained with carboxyfluorescein succinimidyl ester (CFSE) and 4′,6-diamidino-2-phenylindole (DAPI). Briefly, cell cultures were incubated in 5 µM CFSE in PBS for 15 minutes at 37°C. CFSE solution was removed and cultures washed three times in PBS. Cells were counterstained with DAPI for 10 minutes at room temperature. Alternatively, for CFTR immunostaining human amnion epithelial cells were fixed in 4% paraformaldehyde in PBS for 15 minutes at 4°C and permeabilized with 0.1% Triton-X-100 in PBS for 5 minutes at room temperature. DAKO Protein Block Serum-Free was used to block non-specific binding (10 minutes at room temperature) and cells were incubated overnight at 4°C in 8 µg/mL human CFTR C-Terminus antibody (#MAB25031, R&D Systems) diluted in DAKO antibody diluent. Alexa Fluor 488 goat anti-mouse IgG (#A-11001, Life Technologies) was used as a secondary antibody at 40 µg/mL for 1 hour at room temperature. Cells were counterstained with DAPI for 10 minutes at room temperature. Imaging was performed with the Leica DMI 6000 Advanced Fluorescence Imaging System (Leica Microsystems Pty Ltd, North Ryde, NSW, Australia).

### Scanning Electron Microscopy

Human amnion epithelial cells were fixed on plastic coverslips in 4% paraformaldehyde in PBS for 15 minutes at 4°C, frozen at −80°C overnight and freeze dried using the Thermo Scientific SuperModulyo Freeze Dryer system (Thermo Fisher Scientific). Samples were gold sputter coated using the Hummer sputtering system (Anatech Ltd, Battle Creek, MI, USA). Scanning Electron Microscopy (SEM) was performed using the Hitachi S-2600N Scanning Electron Microscope (Hitachi High Techologies, Tokyo, Japan) under high vacuum (HV) between 100x and 500x magnification.

### Soft X-ray Microscopy

A single cell solution of hAECs were obtained by disassociation with 0.05% trypsin for 5 minutes. Cells were fixed in 4% paraformaldehyde in PBS for 15 minutes at 4°C and permeabilized with 0.1% Triton-X-100 in PBS for 5 minutes at room temperature. Cells were incubated with rabbit anti-CFTR primary antibody at 1∶1000 (Cell Signaling #2269, Invitrogen) and then with 1∶1000 Alexa Fluor 488 FluoroNanogold-conjugated secondary antibody (Molecular Probes #A-24922, Invitrogen), each for 1 hr at room temperature. Visualisation of gold particles was enhanced using the LI Silver enhancement kit (Invitrogen). X-ray datasets were acquired using the XM-2 soft X-ray microscope operated by the National Center for X-ray Tomography on beamline 2.1 at the Advanced Light Source (ALS), Lawrence Berkeley National Laboratory, Berkeley, CA, USA. The XM-2 soft X-ray microscope is designed to combine visualization of detailed cellular structure with the location of molecules tagged with gold nanoparticles in cryogenically frozen biological samples with a spatial resolution of approximately 50 nm [11]. Cells were placed inside thin-walled, glass capillary tubes by gentle centrifugation and the tubes subsequently coated with gold nanoparticles, which served as fiducial markers. Cells were rapidly frozen and imaging performed with the specimens in an atmosphere of liquid nitrogen cooled helium gas using photon energies just below the oxygen edge (517 eV), corresponding to an X-ray wavelength of 2.4 nm. Each dataset was comprised of a series of 90 flat field images, acquired sequentially every 2 degrees through 180 degrees of rotation. Images were normalised against a bright field and manually aligned in IMOD 4.1 software (Boulder Laboratory for 3D Electron Microscopy, University of Colorado, Boulder, CO, USA) using the gold fiducial markers. Tomographic reconstructions were performed using iterative reconstruction methods [12], [13] with volume rendering achieved using AmiraDev 3 software (TGS Inc, San Diego, CA, USA). The colour and transparency of the colour-map for the rendered volume was adjusted to visualize cell nuclei (blue) while emphasizing the gold-labeled silver-enhanced antibodies (red).

### Functional Chloride Channel Assay

Plastic cultured hAECs were maintained in either SAGM or DMEM/F12 with 10% FBS for up to 28 days without passage. Primary fetal rat lung epithelial (FRLE) cells were provided as a gift from Dr Annie McDougall, Monash Institute of Medical Research (Clayton, VIC, Australia) and were used as a positive control. Functional analysis of CFTR was performed using the colorimetric technique of Tang and Wildey [14] on the total population (both monolayer and plastic adherent cells). Briefly, CFTR activation by forskolin was determined using iodine as an indicator of chloride channel function. CFTR also conducts other negatively charged ions, including Br^−^, NO3^−^ and I^−^ [15]. Both the stable and radioactive isotopes of I^−^ have been utilized for functional chloride channel assays. In our experiment, the stable isotope of I^−^ was used to measure iodine efflux, expressed as a function of forskolin concentration. 96 well plates containing cells were incubated with iodine-loading buffer (150 mM NaI, 2 mM CaCl_2_, 0.8 mM NaH_2_PO_4_, 1 mM of MgCl_2_, and 5.4 mM of KI; adjusted to pH 7.4 with 1 N sodium hydroxide) for 4 hrs at 37°C. After washing, CFTR was activated through addition of forskolin at concentrations ranging from 0–100 µM resuspended in Dulbecco's phosphate-buffered saline (DPBS) for 5 min, with or without the presence of CFTR-inhibitor CFTR-172 (Sigma-Aldrich). Supernatant iodine concentration was measured with a modified Sandell-Kolthoff reaction. Briefly, 100 µL supernatant samples were mixed with 100 µL of detection buffer I (19.8 mg/L arsenic(III) acid, 25 mg/L ammonium chloride, 1.25% (v/v) ammonia in deionized water, adjusted to pH 7 with sulfuric acid) and 100 µL detection buffer II (20 g/L of ammonia Ce(IV)-sulfate with 5% (v/v) sulfuric acid in deionized water). The mixture was incubated at room temperature for 30 minutes before the OD405 reading with SpectraMax M5 microplate reader (Molecular Devices). Supernatant iodine concentration was calculated from a standard curve and CFTR channel function was calculated and plotted as supernatant iodine concentration vs forskolin concentration. The detection range of iodine for this method was determined to be 0.1 µM–1 µM (data not shown).

### X-ray Fluorescence Microscopy

hAECs were maintained on silicon nitride membrane windows (Silson, Blisworth, Northampton, England) in SAGM for 28 days without passage. Primary fetal rat lung epithelial (FRLE) cells were utilized as a positive control. Cells were incubated in iodine-loading buffer (150 mM NaI, 2 mM CaCl_2_, 0.8 mM NaH_2_PO_4_, 1 mM of MgCl_2_, and 5.4 mM of KI; adjusted to pH 7.4 with 1 N sodium hydroxide) and CFTR was activated through addition of 100 µM forskolin in DPBS as described above. After the cells had been treated windows were washed in a 0.2 M ammonium acetate solution in DPBS, and excess liquid removed by gently touching to a paper towel. Silson windows were then flash frozen in liquid nitrogen-cooled isopentane and immediately transferred to a 0.5 mL Eppendorf tube on dry ice. Windows were freeze-dried overnight and stored in a desiccator at 4°C.

The distribution of intracellular elements was mapped at the X-ray Fluorescence Microscopy beamline of the Australian Synchrotron [16]. A beam of 9.9 keV x-rays was focussed to a spot of approximately 1 µm diameter using a Fresnel zone plate (160 µm diameter, 100 nm outermost zone width, Au, 1600 nm thick). Cells were step-scanned through this focus at 1 µm intervals with a dwell of 1 s. X-ray fluorescence spectra were recorded at each position using a Vortex-EM SDD detector located in the horizontal plane but oriented at 107 degrees to the beam. Full spectra were fitted using MAPS [17] to determine elemental distributions across the scanned areas.

## Results

### Induction of CFTR Gene and Protein Expression in hAECs

**Figure 1:**
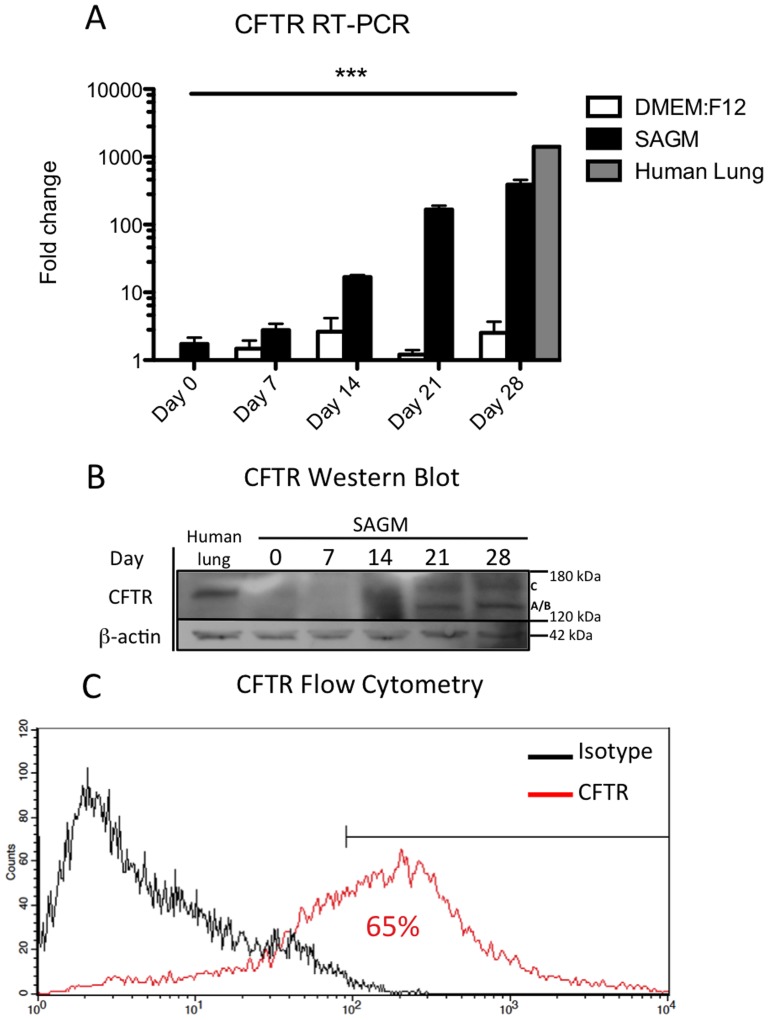
SAGM-cultured hAECs express CFTR gene and protein. CFTR expression in hAECs cultured in DMEM:F12 (white bars) and hAECs cultured in Small Airway Epithelial Growth Medium (SAGM) (black bars) for 7, 14, 21 and 28 days (A) (n = 3). hAECs cultured in SAGM for 28 days had a 389.4±70.0 fold increase in CFTR gene expression (*p≤*0.001), (error bars represent SD). hAECs cultured in DMEM:F12 did not show a significant increase in CFTR gene expression. Human lung total RNA was utilized as a positive control for this study (grey bar). Western blot analysis of CFTR protein expression of freshly isolated hAECs or hAECs cultured in SAGM for 7, 14, 21 and 28 days (B). Both the immature (band A/B) and glycosylated (band C) forms of CFTR were detected in hAEC samples after culture in SAGM for 21 and 28 days (Figure 1B). Only the glycosylated band C was detected in our human lung positive control sample. Flow cytometry analysis of CFTR expression in SAGM cultured hAECs demonstrated that approximately 65% of hAECs expressed the CFTR protein (C) (n = 3).

To determine *CFTR* induction in hAECs we measured gene expression in both freshly isolated hAECs and after culture in either DMEM:F12 or SAGM for 7, 14, 21 and 28 days (n = 3 cell isolations for each timepoint). We did not detect significant *CFTR* gene expression in either freshly isolated cells or following maintenance in DMEM:F12. When cultured in SAGM, *CFTR* gene expression in hAECs increased 390±70 fold over 28 days (*p≤*0.001) (Figure 1A). Human lung total RNA was utilized as a positive control. We then measured CFTR protein by Western blot in freshly isolated hAECs and hAECs cultured in SAGM for up to 28 days. Human lung protein was used as a positive control. Consistent with the gene expression data, CFTR protein was not detected in primary hAECs but became detectable after culture in SAGM for 21 and 28 days (Figure 1B). Both the immature (band A/B) and fully glycosylated (band C) forms of CFTR were detected in hAEC samples, while only a single glycosylated band was detected in our human lung positive control sample. Flow cytometric analysis showed that about 65% of hAECs cultured in SAGM for 28 days were positive for the CFTR protein (Figure 1C).

### Differentiation of hAECs Induces 3D Structures

**Figure 2:**
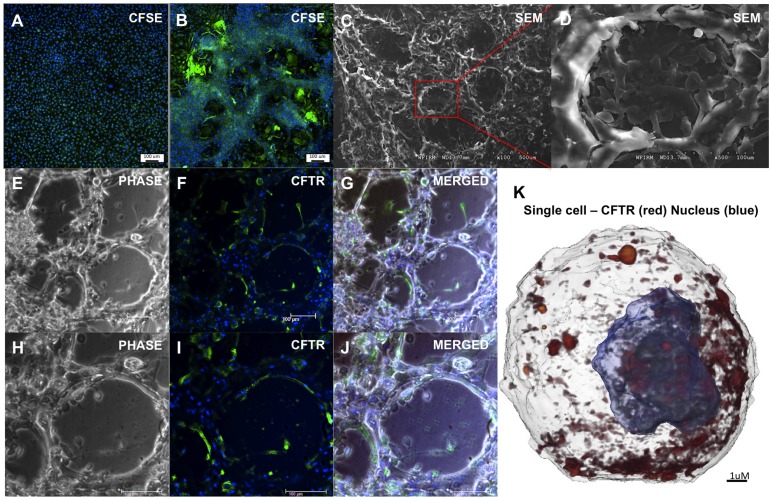
SAGM-cultured hAECs form 3-dimensional structures positive for CFTR. hAECs cultured in DMEM/F12 visualized with fluorescent dyes (CFSE (green) and DAPI (blue)) remained as a confluent, plastic-adherent monolayer (A) (n = 3). hAECs cultured in SAGM for 28 days formed layers of cells clustered into a honeycomb-like morphology growing above a plastic-adherent hAEC monolayer (B) (n = 3). Scanning Electron Microscopy (SEM) analysis of the honeycomb-like 3D structures reveal close interactions between the plastic adherent hAECs and hAECs within the 3D honeycomb-like structures (C (100x) and D (500x)) (n = 3). CFTR expressing cells were found within all 3D structures observed in culture (E–G) and appeared to localize to the edges of the 3D cell clusters (H–J) (n = 3). X-ray tomographic image of the subcellular distribution of CFTR protein using X-ray tomography (K) (n = 4). Shown is a single hAEC from a culture of hAECs after 28 days in SAGM. We were able to visualize cell nuclei (blue), and localize the gold-labeled, silver-enhanced CFTR antibodies (red). CFTR was abundant on the cell membrane, and localized to an area covering approximately half of the plasma membrane.

We visualized cultured hAECs using carboxyfluorescein succinimidyl ester (CFSE) and 4′,6-diamidino-2-phenylindole (DAPI). We observed that, while hAECs cultured in DMEM:F12 remained as a confluent monolayer (n = 3, Figure 2A), hAECs maintained in SAGM formed 3-dimensional (3D) honeycomb-like structures of clustered cells growing above a plastic adherent hAEC monolayer (n = 3, Figure 2B). To further characterize these 3D structures we used scanning electron microscopy (SEM), confirming the 3D honeycomb-like structures formed by hAECs in SAGM (Figure 2C and D).

### Localization and Polarization of CFTR Expressing Cells

Next, to localize the cells that expressed CFTR within the 3D structures we labelled cells with an anti-CFTR primary antibody and an Alexa Fluor® 488-conjugated secondary antibody. We found CFTR expressing cells within the 3D structures (Figure 2E–G), mainly localized to the “luminal” borders of these clusters (Figure 2H–J). To determine the distribution of CFTR protein within the cell membrane of single cells harvested from confluent hAEC cultures maintained in SAGM for 28 days (n = 4), we used synchrotron soft X-ray microscopy to detect gold labelled CFTR. In these cells CFTR was abundant on the cell membrane and was localized to an area covering approximately half of the plasma membrane (Figure 2K and Video S1– single cell shown), suggestive of polarization of hAECs during culture and differentiation. CFTR protein was not detected on membranes of single cells analysed from undifferentiated hAEC cultures (n = 4) (data not shown).

### Functional Assessment of CFTR in Differentiated hAECs

**Figure 3:**
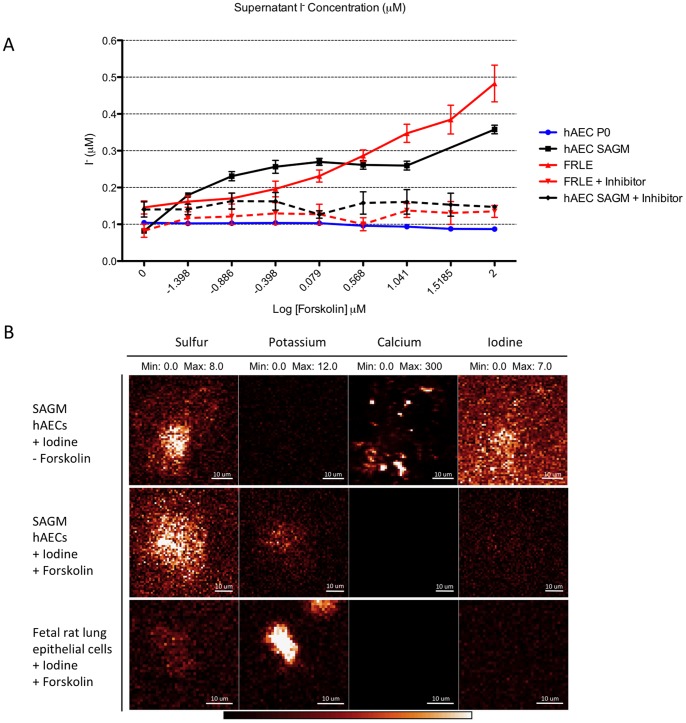
SAGM-cultured hAECs produce functional CFTR ion channels. Supernatant iodine concentration was measured and CFTR channel function was calculated and plotted as supernatant iodine concentration vs forskolin concentration (A) (error bars represent SD, n = 9). hAECs cultured in SAGM demonstrated forskolin-activated CFTR function. Treatment of cultures with CFTR-inhibitor CFTR-172 reversed forskolin activation of CFTR function. Fetal rat lung epithelial (FRLE) cells were utilized as a positive control in this study and displayed similar CFTR stimulation and inhibition as observed in SAGM-cultured hAECs. We did not detect any significant forskolin-activated CFTR function in undifferentiated hAECs. 2D elemental mapping of the forskolin-activated flux of iodine across the cell membrane as well as 2D elemental maps for sulfur, potassium and calcium (B) supported the observed forskolin-activated ion flux across the membrane (n = 3).

To assess whether the increased CFTR protein production induced in the hAECs cultured in SAGM was functional we used a colorimetric technique [14]. We observed that hAECs cultured in SAGM demonstrated forskolin-activated CFTR function that was blocked by a specific CFTR inhibitor (Figure 3A). In these cells, forskolin activated CFTR with a half maximal effective forskolin concentration (EC_50_) of 2.9±1.2 µM (n = 9), compared to an EC_50_ of 0.95±1.2 µM for fetal rat lung epithelial (FRLE) cells. Treatment of FRLE cells or SAGM cultured hAECs with the CFTR inhibitor CFTR-172 resulted in an inhibition of forskolin activation of CFTR function and a reduction in supernatant NaI concentration (Figure 3A). We did not detect any forskolin-activated CFTR function in undifferentiated hAECs (n = 9), consistent with the lack of both CFTR mRNA expression and protein immunolocalization in these cells. To confirm forskolin activation of CFTR in SAGM cultured hAECs we used 2D elemental mapping of forskolin-activated flux of iodine across the cell membrane utilizing X-ray fluorescence microscopy (XFM). SAGM cultured hAECs incubated with forskolin, following culture in iodine loading buffer contained less intracellular iodine than in SAGM cultured hAECs not exposed to forskolin consistent with an efflux of iodine through the chloride channel (Figure 3B) and out of the cell. We also observed that hAECs contained more intracellular potassium (K^+^) and less intracellular calcium (Ca^++^) following incubation with forskolin.

## Discussion

CFTR expression and function is essential for normal respiratory airway function and is defective in individuals with cystic fibrosis. With a view to developing a cell-based therapy for cystic fibrosis we have shown that human amnion epithelial cells (hAECs) can be induced to express functional cystic fibrosis transmembrane conductance regulator (CFTR) *in vitro* by culture in media designed for lung cell differentiation. This observation supports the development of hAECs as a potential cell source for future CF therapy.

We have previously reported that hAECs can be induced to produce surfactant proteins A, B, C and D and contain lamellar body–like organelles both *in vitro* and *in vivo* [10]. In the current study we have extended those observations by showing that hAECs can be induced to produce CFTR. That primary hAECs do not express the *CFTR* gene or produce CFTR protein was not surprising as these cells do not express other respiratory tract cell markers such as the surfactant genes either, doing so only after differentiation [10]. However, in a recent study of mesenchymal and epithelial cells derived from term amniotic membrane *CFTR* gene expression was observed in both amniotic mesenchymal cells and hAECs, albeit only after nested RT-PCR [18], although expression declined rapidly in culture. Whether hAECs also expressed CFTR protein was not reported and no information about purity of cell populations was provided. The current study suggests that primary hAECs do not express *CFTR* gene or make the protein, doing so only after extended culture in SAGM. Interestingly, when mesenchymal cells are cultured in direct contact with lung airway cells *CFTR* gene expression is induced within 6 days [18]. That induction of *CFTR* expression was much faster than we have observed in hAECs in SAGM but is consistent with the induction of surfactant protein gene expression by hAECs *in vivo* [10]. This suggests that differentiation of placental cells, whether MSCs or hAECs, into lung lineages may be most efficient when in direct contact with lung cells as would happen with *in vivo* engraftment. If this is the case then partial differentiation *in vitro* before administration into the airway as a therapy for CF may not be necessary, as was the case for hAEC administration following acute injury [10].

The observation that hAECs formed 3 dimensional cell aggregates following culture on plastic surfaces in SAGM was new. In our previous work when we cultured hAECs in SAGM to induce surfactant protein expression we did not observe the formation of such structures. However, in those experiments hAECs were grown on glass coverslips or on transwells coated with collagen type IV. hAECs have previously been utilized as feeder cells for limbal epithelial progenitor cells [19] and we postulate that the monolayer of plastic adherent hAEC may function as a feeder layer to support the growth and differentiation of cells within the 3D structures. In this study we did not compare lung-specific marker expression in hAECs maintained in different culture surfaces and further experiments will be required to characterize the culture surface properties that lead to the formation of these 3D structures. Further, we did not assess whether formation of the 3D structures is necessary for CFTR expression but it was interesting that the distribution of CFTR expressing cells was quite specific, limited to cells on the “lumenal” surfaces of the structure, as might be expected of CFTR expressing cells in the airway.

In addition to localization of the CFTR expressing cells, we were interested in localisation of CFTR within individual cells. In the airway, CFTR is localized to the apical plasma membrane of ciliated cells, a localization that is disrupted by CFTR mutations [20], [21]. Using synchrotron soft X-ray microscopy we observed a polarized CFTR distribution on the membrane of single hAECs obtained from dissociated SAGM cultures. As CFTR expressing cells appeared to be localized within 3D structures, hAECs may be induced to express CFTR on the outer, “apical”, surface of the cell to allow effective transport of ions between cells within the structures and the surrounding media in a manner similar to ion channels in polarized airway cells. Air-liquid interface culture is a method commonly used to generate differentiated, polarized cell populations *in vitro* and could be used to induce CFTR expression in hAEC cultures. However, with the limitations of scale up of air-liquid interface culture conditions if CFTR expressing cells, as opposed to primary cells, are required for clinical therapies, future studies may be better focussed on improving the efficiency of 3D structure formation *in vitro* and on developing methods to identify and purify CFTR-expressing hAECs.

An important function of CFTR in airway epithelial cells is the regulation of ion and water flows across airway surfaces. It is a lack of this regulation that leads to the tenacious mucus and respiratory consequences of cystic fibrosis. Forskolin activates CFTR via protein kinase A (PKA) dependent phosphorylation of the regulatory domain of CFTR [22], [23]. Consistent with the production of both *CFTR* mRNA and protein, we observed that hAECs cultured in SAGM demonstrated forskolin-activated CFTR function while treatment of cultures with the CFTR-inhibitor CFTR-172 blocked forskolin activation of CFTR function, as would be expected. Forskolin-activated flux of iodine across the cell membrane was determined using both an established colorimetric technique and by X-ray fluorescence microscopy (XFM). This combined approach not only validated the read-outs of each approach but also allowed us to detect changes in intracellular potassium (K^+^) and calcium (Ca^++^) in forskolin-activated cells. Unfortunately, CFTR interaction with the Na^+^ channels such as EnaC could not be investigated due to the presence of Na^+^ in processing solutions. The observed changes in intracellular potassium (K^+^) and calcium (Ca^++^) are likely to be the result of hAECs maintaining electroneutrality following forskolin-induced chloride ion flux. However, CFTR activation has previously been shown to regulate other ion channels including members of the inward rectifier family of K^+^ channels (Kir) [24] and has complex interactions with regulators of calcium channels as well as with intracellular calcium [25], [26], [27]. Therefore, it is possible that the changes in intracellular K^+^ and Ca^++^ following CFTR activation was secondary to specific activation of other ion channels rather than simply maintenance of electroneutrality. Further studies are required to clarify this. Nonetheless, taken together, these data demonstrate the ability to induce hAECs to express functional CFTR *in vitro.*

In summary, we have shown that by culturing hAECs in SAGM we could induce hAECs to form 3D “alveoli-like” structures and to express functional CFTR ion channels on the luminal aspects of those structures. We have previously demonstrated that hAECs are anti-inflammatory and reduce fibrosis in animal models of acute lung injury [10], [28] and that systemic administration results in significant hAEC engraftment in the lung [10]. We propose that hAECs are a promising source of regenerative cells for cellular therapy for cystic fibrosis. We are now investigating the potential to purify CFTR-expressing cells and are developing methods to achieve significant pulmonary engraftment of hAECs in animal models of cystic fibrosis.

## Supporting Information

Video S1 X-ray tomographic image of the subcellular distribution of CFTR protein using synchrotron soft X-ray microscopy. Shown is a single hAEC from a culture of hAECs after 28 days in SAGM. We were able to visualize cell nuclei (blue), and localize the gold-labeled, silver-enhanced CFTR antibodies (red). CFTR was abundant on the cell membrane, and localized to an area covering approximately half of the plasma membrane (n = 4).
(MOV)
